# Repair-Related Activation of Hedgehog Signaling in Stromal Cells Promotes Intrahepatic Hypothyroidism

**DOI:** 10.1210/en.2014-1302

**Published:** 2014-08-14

**Authors:** Brittany N. Bohinc, Gregory Michelotti, Guanhua Xie, Herbert Pang, Ayako Suzuki, Cynthia D. Guy, Dawn Piercy, Leandi Kruger, Marzena Swiderska-Syn, Mariana Machado, Thiago Pereira, Ann Marie Zavacki, Manal Abdelmalek, Anna Mae Diehl

**Affiliations:** Divisions of Endocrinology, Diabetes, and Metabolism (B.N.B., D.P.) and Gastroenterology (G.M., G.X., A.S., L.K., M.S.-S., M.M., T.P., M.A., A.M.D.) and Departments of Biostatistics and Bioinformatics (H.P.) and Pathology (C.D.G.), Duke University, Durham, North Carolina 27710; and Division of Endocrinology, Diabetes, and Metabolism (A.M.Z.), Brigham and Women's Hospital, Boston, Massachusetts 02115

## Abstract

Thyroid hormone (TH) is important for tissue repair because it regulates cellular differentiation. Intrahepatic TH activity is controlled by both serum TH levels and hepatic deiodinases. TH substrate (T_4_) is converted into active hormone (T_3_) by deiodinase 1 (D1) but into inactive hormone (rT_3_) by deiodinase 3 (D3). Although the relative expressions of D1 and D3 are known to change during liver injury, the cell types and signaling mechanisms involved are unclear. We evaluated the hypothesis that changes in hepatic deiodinases result from repair-related activation of the Hedgehog pathway in stromal cells. We localized deiodinase expression, assessed changes during injury, and determined how targeted manipulation of Hedgehog signaling in stromal cells impacted hepatic deiodinase expression, TH content, and TH action in rodents. Humans with chronic liver disease were also studied. In healthy liver, hepatocytes strongly expressed D1 and stromal cells weakly expressed D3. During injury, hepatocyte expression of D1 decreased, whereas stromal expression of D3 increased, particularly in myofibroblasts. Conditionally disrupting Hedgehog signaling in myofibroblasts normalized deiodinase expression. Repair-related changes in deiodinases were accompanied by reduced hepatic TH content and TH-regulated gene expression. In patients, this was reflected by increased serum rT_3_. Moreover, the decreases in the free T_3_ to rT_3_ and free T_4_ to rT_3_ ratios distinguished advanced from mild fibrosis, even in individuals with similar serum levels of TSH and free T_4_. In conclusion, the Hedgehog-dependent changes in liver stromal cells drive repair-related changes in hepatic deiodinase expression that promote intrahepatic hypothyroidism, thereby limiting exposure to T_3_, an important factor for cellular differentiation.

Thyroid hormone (TH) controls cellular metabolism and differentiation. Its local activity must be appropriately regulated to accomplish organogenesis during development and tissue repair during adulthood (1). Tissue TH activity is regulated by deiodinases (D) 1, 2, and 3. In the liver, D1 converts substrate T_4_ to biologically active T_3_, whereas D3 inactivates T_4_ and T_3_ by converting them to more metabolically inert products, rT_3_ and diiodothyronine (T_2_). rT_3,_ in turn, is cleared by D1-catalyzed conversion to T_2_ (Figure 1A) (2). Both activating and deactivating deiodinases work in concert to maintain TH homeostasis. Hence, alterations in deiodinase balance can affect downstream tissue-specific TH signaling and local action. During early embryogenesis, for example, D3 is highly expressed to limit cellular exposure to T_3_ (3, 4). After birth, D3 expression declines and expression of D1 and D2 increases, allowing for T_3_-induced tissue maturation and cellular differentiation (4). The reciprocal developmental functions of activating and inactivating deiodinases explain why D3 knockout mice exhibit growth restriction, neonatal mortality, and abnormal thyroid status (4). Interestingly, selective reexpression of D3 occurs during adult tissue injury, as evidenced by the up-regulation of hepatic D3 after partial hepatectomy, induction of D3 in cardiac muscle after myocardial infarction, and increased D3 expression in injured brain and skeletal muscle (5–7). Conversely, hepatic D1 activity generally falls in many chronic illnesses (8). Because liver D1 plays a significant role in regulating peripheral conversion of T_4_ to T_3_, this contributes to the decreases in liver and systemic T_3_ levels that occur in the euthyroid sick syndrome. Despite considerable evidence that tight control of TH homeostasis is necessary for normal tissue growth and differentiation and the acknowledged importance of deiodinases in regulating local TH activity, the mechanisms that control D1 and D3 during adult liver repair remain poorly understood.

**Figure 1.**
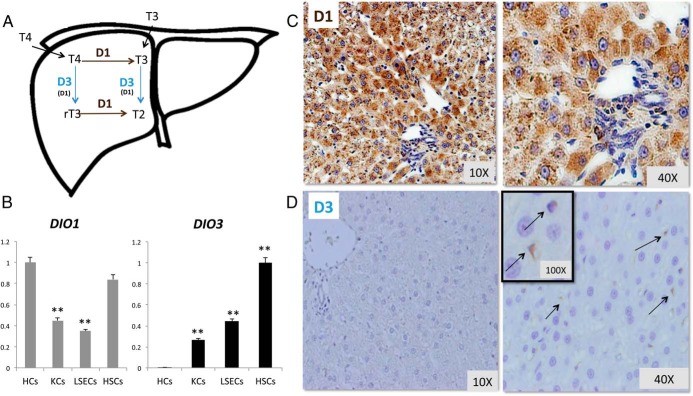
Differential expression of D1 and D3 in different liver cell types. A, Diagram demonstrating respective actions of D1 and D3. B, qRT-PCR analysis of *DIO1* and *DIO3* mRNAs in primary mice hepatocytes (HC), HSCs, KCs, and LSECs. *DIO1* and *DIO3* levels are normalized to the expression in HCs and HSCs, respectively; mean ± SEM are graphed. **, *P* < .01. Immunohistochemistry for D1 (C) and D3 (D) protein in livers of representative healthy adult rats are shown (magnification, ×10 and ×40). The arrows are pointing to minimal basal D3 expression observed in nonparenchymal cells in uninjured liver.

Evidence that D3 expression predominates during adult liver regeneration suggested to us that this enzyme might localize in stromal cell types because these cells accumulate in response to factors produced during liver injury. We further theorized that injury-related Hedgehog pathway activation in the liver stromal compartment triggers reacquisition of a more fetal-like pattern of hepatic deiodinase expression because developing tissues in early embryos are particularly enriched with D3, and certain developmental morphogenic signaling pathways, such as Hedgehog, must become locally reactivated in hepatic stromal cells for injured adult livers to regenerate (9). To evaluate this hypothesis, we compared expression of D1 and D3 in various types of rodent liver cells and liver tissue before and after adult liver injury and assessed how the targeted manipulation of Hedgehog signaling in liver stromal cells influenced hepatic deiodinase expression, TH content, and tissue TH action. Humans with chronic liver disease were also studied to verify the translational applications of our findings. Our results confirm the hypothesis that the injured liver is in a state of intrahepatic hypothyroidism, identifying previously unsuspected roles for hepatic stromal cells and the canonical Hedgehog pathway as key regulators of TH homeostasis during adult liver injury. As such, our findings provide novel insight into both the mechanisms for, and the implications of, the euthyroid sick syndrome.

## Materials and Methods

### Rodent liver cell isolation and culture.

Primary hepatocytes (HCs), Kupffer cells (KCs), hepatic stellate cells (HSCs), and liver sinusoidal epithelial cells (LSECs) were isolated from male C57/BL6 mice (Jackson Laboratories) and male Sprague Dawley rats (Charles River Laboratories) (10–13). The rat 8B stellate cell line was cultured as described (14), treated with Sonic Hedgehog ligand (SHH-L; 10 ng/mL) or vehicle (sterile PBS + 0.1% BSA), and harvested after 24 hours.

### Animal models of liver injury

To assess the effect of liver injury on deiodinases, rats (n = 18) underwent sham surgery or bile duct ligation (BDL), and were killed on days 3, 7, or 14. To address the role of Hedgehog signaling in regulating hepatic deiodinases, adult male double transgenic (DTG) αSMA-CreERT2 × SMO/flox homozygote mice that have previously been fully characterized (15) were subjected to sham surgery (n = 8 mice) or BDL (n = 22 mice/group). Tamoxifen (TMX) was administered postoperatively to activate Cre recombinase and conditionally disrupt Hedgehog signaling (15). Results were compared with vehicle-treated controls. Livers were harvested 14 days after BDL. The Animal Care and Use Committee and the Duke University Institutional Animal Care and Use Committee approved animal care and surgical procedures.

### Human studies

Liver and serum samples from 2 cohorts of patients in our Non-Alcoholic Fatty Liver Disease (NAFLD) Clinical Database and Biorepository were analyzed for TH content and deiodinase expression. Among the discovery cohort (n = 70 patients), 42 individuals had histologically proven mild NAFLD fibrosis [stage 0–1 hepatic fibrosis by the Nonalcoholic Steatohepatitis Clinical Research Network scoring system (16)], and 28 patients had advanced NAFLD fibrosis (stage 3–4 hepatic fibrosis). The 2 groups were matched for gender, age, and body mass index (kilograms per square meter) and analyses corrected for potential confounders. A second validation cohort (n = 30 mild, n = 30 advanced) was separately analyzed.

### Assays

#### mRNA isolation and analysis

Total RNA was extracted from rodent liver tissue/cells and analyzed in triplicate by quantitative RT-PCR (qRT-PCR) as described (see Supplemental Methods). Liver RNA from the discovery patient cohort was analyzed by microarray analysis; differences in TH-responsive protein (*THRSP*; a TH inducible gene) were determined using *limma* (R/Bioconductor statistical package), followed by qRT-PCR validation (17).

#### Western blotting

Protein lysates were prepared with radioimmunoprecipitation assay and/or Laemmli buffer, separated by SDS-PAGE, and transferred to nylon membranes for detection by the Bio-Rad Laboratories ChemiDoc MP imaging system.

#### Flow cytometry

D1 or D3 proteins were evaluated in primary rat HSCs using methods of flow cytometry as previously described (18).

#### Liver histology

All human liver biopsy specimens were stained with hematoxylin-eosin and Masson's trichrome stains and were graded and staged by a hepatopathologist (C.D.G.) according to the Nonalcoholic Steatohepatitis Clinical Research Network Scoring System (16).

#### Liver immunohistochemistry (IHC) and morphometry

Rodent and human liver specimens were prepared for IHC as described (18).

#### Intrahepatic TH measurements

Concentrations of T_4_ and T_3_ were determined in homogenized whole livers from male rats (n = 6) and male DTG αSMA-Cre × SMO/flox homozygote mice (n = 9) treated with sham surgery or BDL surgery using an Agilent 6410 triple quadruple tandem mass spectrometer (liquid chromatography and tandem mass spectrometry, LC/MS-MS) using established methods (19).

#### Serum TH measurement

Rodent serum TH levels were measured using established methods (17). Human serum TH measures including the following: TSH, free T4 (fT_4_), free T3 (fT_3_), and rT_3_; fT_3_ to rT_3_ and fT_4_ to rT_3_ ratios were obtained on serum collected from patients on the same day as the liver biopsy.

### Statistical analysis

Significance (*P* < .05) was assessed by a Student's *t* test, Pearson's χ^2^ test, Wilcoxon rank-sum test, and/or univariate/multivariate analysis where appropriate. All analyses were done using JMP statistical software version 7.0 (SAS Institute Inc).

See Supplemental Tables 1–6, Supplemental Figure 1, and Supplemental Methods for complete details.

## Results

### Differential expression of D1 and D3 in hepatocytes and liver stromal cells

Primary HCs and various types of liver stromal cells (eg, HSCs, KCs, and LSECs) from healthy, adult mice were analyzed for mRNA expression of *DIO1* and *DIO3*, the main deiodinases that regulate T_4_ activation/deactivation in adult liver (Figure 1A). Although all cell types expressed *DIO1* mRNA, levels of this transcript were particularly enriched in hepatocytes, which expressed high levels of *DIO1*, but were barely detectable *DIO3* (Figure 1B). In contrast, the expression of *DIO1* and *DIO3* transcripts was more balanced in liver stromal cells, with HSCs demonstrating the highest levels of *DIO3* mRNA among the various liver cell types (Figure 1B). In healthy liver, IHC confirmed ubiquitous strong expression of D1 protein but a much weaker expression of D3 protein that was mainly localized in stromal cells [Figure 1C (×10 and ×40) and Figure 1D (×10 and ×40)].

### Liver injury provokes reciprocal changes in net hepatic expression of D1 and D3

**Figure 2.**
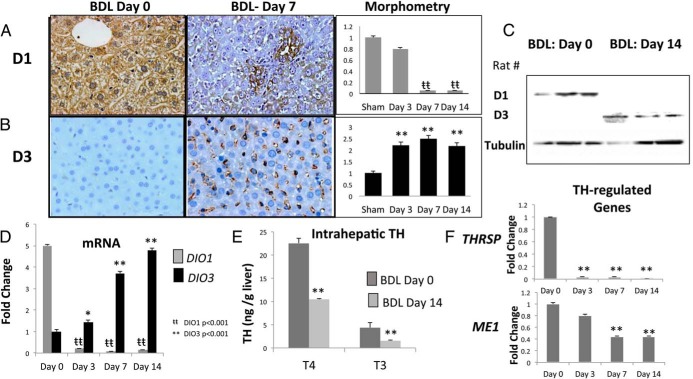
Liver injury induces reciprocal changes in D1/D3 expression and intrahepatic hypothyroidism. Immunohistochemistry for D1 (A) and D3 (B) in representative rats on days 0 and 7 after BDL is shown. Morphometric analysis of all rats (n = 3 per group per time point); results were normalized to data from sham-operated controls (n = 3) on the day of BDL (day 0) and graphed as mean ± SEM. *, *P* < .05; **, *P* < .01. C, Representative Western blot. D, qRT-PCR analysis of *DIO1* and *DIO3* mRNA expression. *, *P* < .05; **, *P* < .01. E, Intrahepatic T_4_ and T_3_ concentrations on days 0 and 14 after BDL. **, *P* < .01. F, qRT-PCR analysis of T_3_-responsive genes *THRSP* and *ME1*. **, *P* < .01.

The pattern of deiodinase expression changed dramatically during liver injury. After bile duct ligation in rats, for example, staining for D1 virtually disappeared, while D3 protein was strongly increased (Figure 2, A and B). Western blot analysis of whole liver protein confirmed the immunohistochemistry data (Figure 2C). Changes in D1 and D3 protein expression generally paralleled changes in the respective whole liver mRNA levels (Figure 2D). Because TH substrate (T_4_) is converted to the more biologically-active TH (T_3_) by D1, whereas D3 converts T_4_ into a biologically-inert TH (reverse T_3_, rT_3_) and further inactivates T_3_ by conversion to T_2_ (Figure 1A), injury-related changes in D1 and D3 are predicted to limit hepatic accumulation of both bioactive forms of TH (ie, T_4_ and T_3_). Indeed, liquid chromatography and tandem mass spectrometry measurement of TH concentrations before and after BDL confirmed that intrahepatic and serum concentrations of both T_4_ and T_3_ decreased significantly by day 14 post-BDL (Figure 2E and Supplemental Table 5), a time when there is considerable cholestatic liver injury and fibrosis (Supplemental Figure 1). T_4_ levels fell significantly more in the liver than in the serum after BDL (53.7% vs 20.7%, *P* < .01), suggesting that increased hepatic D3 was important in regulating liver TH content during liver injury. To determine whether these changes in TH concentrations were accompanied by changes in hepatic TH activity, we evaluated expression of representative TH-regulated genes. After BDL, mRNA levels of *ME1* and *THRSP* (TH responsive genes) both decreased significantly (Figure 2F). Thus, injured liver tissue reacquires a deiodinase expression profile that is reminiscent of developing tissue in which there is limited exposure to TH.

### Stromal cells become the dominant deiodinase-expressing cells in injured liver

**Figure 3.**
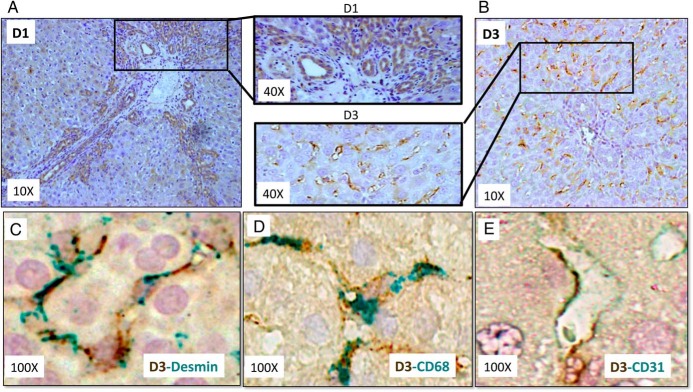
Redistribution of deiodinase expression in injured livers. Immunohistochemistry for DIO1 (A) and DIO3 (B) on day 14 after BDL are shown (magnification, ×10 and ×40). Costaining for D3 (brown) and desmin (green), a stellate cell marker (C), CD68, a macrophage marker (green) (D), and CD31, a marker of liver sinusoidal endothelial cells (green) (E) are shown (magnification, ×100).

To determine whether the observed changes in deiodinase expression reflected changes in residual resident liver cells, as opposed to nonspecific effects caused by liver cell loss and/or infiltrating inflammatory cells, additional immunostaining was done (Figure 3). Surprisingly, the types of liver cells producing deiodinases changed dramatically after liver injury. In injured livers, hepatocytes were no longer the dominant D1-expressing cell. Rather, D1 accumulated in ductular type cells (Figure 3A). D3 protein remained in stromal cells (Figure 3B) and mainly colocalized with markers for HSCs (desmin, Figure 3C), KCs (CD68, Figure 3D), and LSECs (CD31, Figure 3E). Desmin, CD68, and CD31 costains were performed by IHC and revealed that, although stellate cells were the major producers of D3 after injury (comprising >60% of D3 expressing cells), approximately 30% of D3-positive cells were CD68 positive and 10% were CD31 positive. In both instances, CD68- and CD31-producing cells increased in a time-dependent manner after BDL injury, with most cells seen by day 7. Hence, the accumulation of these stromal cells likely contributed to the injury-related shift from D1 to D3 that was demonstrated by analysis of whole liver mRNA and protein (Figure 2, A–D). Thus, liver injury recapitulates the fetal-like state of D3 predominance due not only to a loss of D1 in hepatocytes but also because D3 is dramatically increased in the hepatic stromal compartment.

### Activated hepatic stellate cells are major sources of D3 during liver injury

**Figure 4.**
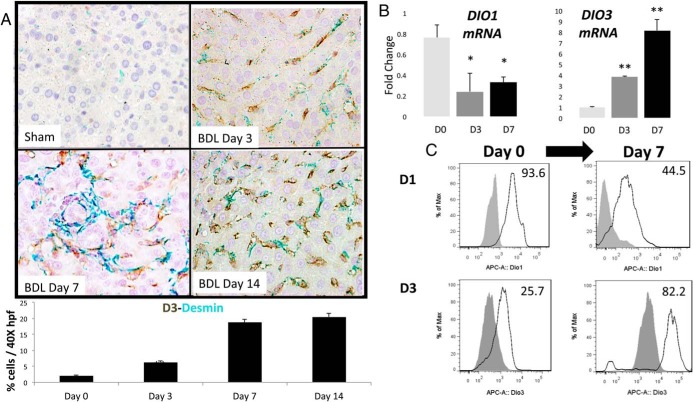
Myofibroblastic hepatic stellate cells are major sources of D3. A, Costaining for D3 (brown) with desmin (green) in representative sham-operated rats and rats at different time points after BDL. Double-positive cells were counted, expressed as a percentage of cells per high-power field (HPF), and graphed as mean ± SEM. **, *P* < .01. B, qRT-PCR analysis of *DIO1* and *DIO3* mRNA at different times during culture of primary rat stellate cells. *, *P* < .05; **, *P* < .01 vs day 0. C, Fluorescence-activated cell sorting analysis of primary stellate cells is shown. Numbers of D1- or D3-positive cells are demonstrated relative to respective IgG-stained controls.

Among liver stromal cells, injury-activated HSCs play particularly pivotal roles in hepatic wound healing/regeneration. In addition to becoming a major source of fibrogenic myofibroblasts, activated HSCs produce various hepatocyte growth factors, chemokines, and mediators of angiogenesis, and there is strong experimental evidence that inhibiting HSC activation drastically impairs liver regeneration (20, 21). Double immunostaining for desmin, a stellate cell-specific marker (22), and D3 confirmed that many of the D3-expressing cells that accumulated after BDL were HSCs (Figure 4A), raising the possibility that HSC might up-regulate their expression of D3 as they activate during injury. To evaluate this more directly, we compared D3 expression in primary HSC at different time points during culture-induced activation. Both *DIO3* mRNA (Figure 4B) and D3 protein (Figure 4C) levels increased as quiescent HSCs became myofibroblasts. This activation-related induction of D3 expression was accompanied by a reduced expression of DIO1 mRNA and D1 protein (Figure 4, B and C). Thus, HSC activation is characterized by reciprocal changes in the expression of *DIO1* and *DIO3*, with the resultant predominance in *DIO3* predicted to reduce accumulation of biologically-active TH.

### Hedgehog signaling in HSC-derived myofibroblasts controls hepatic deiodinase expression and TH homeostasis during liver injury

**Figure 5.**
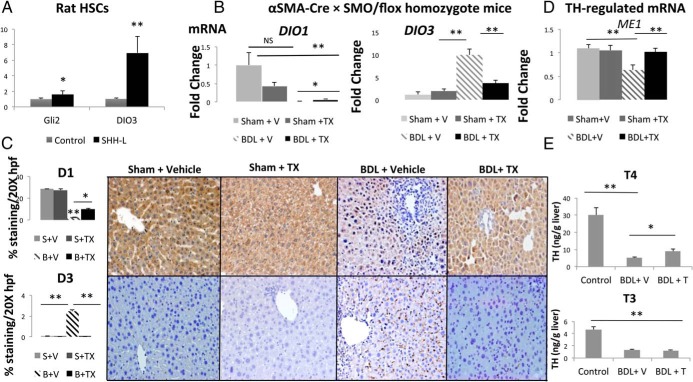
Hedgehog signaling in myofibroblasts controls hepatic deiodinase expression and TH homeostasis during liver injury. A, qRT-PCR analysis of rat stellate cells (HSC 8B) 24 hours after treatment with vehicle or recombinant SHH-L (10 ng/mL). B, qRT-PCR analysis of whole-liver RNA harvested from SMA-Cre × SMO/flox homozygote mice treated with either vehicle (V) or TMX after BDL (n = 11 mice/group) as described in *Materials and Methods*. Results are expressed relative to those in sham-operated vehicle-treated SMA-Cre × SMO/flox homozygote mice (n = 4). **, *P* < .01. C, Morphometric analysis of all mice (**, *P* < .01) and immunohistochemistry of representative mice in each group. D, qRT-PCR analysis of *ME1* mRNA in whole-liver RNA. **, *P* < .01. E, Intrahepatic T_4_ and T_3_ levels measured ± V or TMX. **, *P* < .01.

HSC activation is controlled by canonical Hedgehog signaling. Increasing pathway activity stimulates quiescent HSCs to become myofibroblasts, whereas the reducing pathway activity in myofibroblasts restores the quiescent phenotype (23). To determine whether Hedgehog signaling influences HSC expression of *DIO3*, rat HSC-derived myofibroblasts were treated with recombinant SHH-L, and the expression of *DIO3* was evaluated 24 hours later. Compared with vehicle-treated controls, myofibroblastic HSCs treated with SHH-L demonstrated 50% higher levels of the *SHH*-inducible transcription factor, Glioblastoma 2 (Gli2), and more than 6-fold increased expression of *DIO3* (*P* < .05, and *P* < .01, respectively) (Figure 5A). These findings are consistent with a previous report that *Gli2* directly interacts with the *DIO3* promoter to induce *DIO3* expression in keratinocytes after Sonic Hedgehog exposure (24) and suggest that deiodinase expression in liver myofibroblasts may also be controlled by Hedgehog signaling.

To further investigate this issue, we used αsmaCre-smo^flx/flx^ mice to conditionally delete Smoothened (Smo), an obligate component of the canonical Hedgehog pathway, in myofibroblasts after BDL and compared deiodinase expression, hepatic TH levels, and hepatic expression of TH-regulated genes in mice with and without canonical signaling in HSCs. Previously we reported that treating such αsmaCre-smo^flx/flx^ mice with tamoxifen after BDL deleted Smo from myofibroblastic HSCs and demonstrated that this targeted inhibition of Hedgehog signaling decreased multiple fibrotic and regenerative responses that are typically provoked by cholestatic liver injury (15). Here we show that hepatic TH homeostasis was severely disrupted in the BDL mice that lacked Smo in HSC-derived myofibroblasts compared with BDL controls with intact HSC Hedgehog signaling. Both the induction of *DIO3* and the fall in *DIO1* mRNA that normally follow BDL were partially blocked by disrupting Hedgehog signaling in myofibroblasts (Figures 5B). Surprisingly, changes in deiodinase expression were not limited to the stromal compartment. Immunostaining confirmed that disrupting Hedgehog signaling in myofibroblasts reduced accumulation of D3-positive stromal cells but also revealed relative restoration of D1 expression in hepatocytes (Figure 5C). This finding suggests a previously unsuspected role for stromal cells in regulating net hepatic deiodinase expression, and thereby local TH activity, during liver injury. Because limiting myofibroblast accumulation during liver injury tended to restore the D1-predominant state that is typical of uninjured liver, this might prevent injury-related depletion of intrahepatic TH.

Consistent with that concept, hepatic concentrations of T_4_ were found to be about twice as high in the Smo-deficient (myofibroblast depleted) mice as in control mice 14 days after BDL (8.99 vs 4.99 ng T_4_ per gram of liver, *P* < .05), despite continued euthyroid sick pattern of suppressed serum TH levels after BDL injury in both models (57% and 62% decrease in serum T_4_ in mice after BDL ± TMX compared with sham, respectively). The increase in intrahepatic TH levels was accompanied by an increased hepatic expression of the TH-responsive gene, *ME1* (Figure 5, D and E). Intrahepatic T_3_ concentrations and hepatic expression of the T_3_-responsive gene, *THRSP*, were similar in Smo-deficient mice and controls 14 days after BDL (data not shown). Serum rT_3_ levels increased significantly (*P* = .026) after BDL injury and fell to baseline levels with the addition of TMX and the resultant disruption of Hedgehog signaling in myofibroblasts, paralleling changes in hepatic D3 expression (Supplemental Table 6). Taken together, our findings demonstrate that Hedgehog-responsive myofibroblasts are important regulators of hepatic deiodinase expression during liver injury and are partially responsible for the intrahepatic hypothyroidism that develops after BDL in mice.

### Injury-related changes in hepatic deiodinases parallel hepatic accumulation of Hedgehog-responsive myofibroblasts and contribute to tissue hypothyroidism in humans

Recently we reported that the hepatic gene expression profiles of NAFLD patients with advanced liver fibrosis reflected active tissue remodeling, including relative enrichment with various Hedgehog-regulated genes (17). Here we compared serum TH profiles in these same age-, gender-, and body mass index-matched NAFLD subjects with either advanced or mild fibrosis and found that subjects with advanced NAFLD fibrosis had lower levels of fT_3_ and higher levels of rT_3_ (ie, a reduced fT_3_ to rT_3_ ratio) (Table 1). Indeed, in both the discovery and validation cohorts, the fT_3_ to rT_3_ ratio and the fT_4_ to rT_3_ ratio correlated significantly with the stage of liver fibrosis as determined by multivariate logistic regression analysis. Given that D1 converts T_4_ to T_3_ and then clears rT_3_, whereas D3 converts T_4_ to rT_3_ and clears T_3_, this TH pattern suggests that a switch from D1 predominance to D3 predominance occurs during advanced NAFLD fibrosis in humans.

**Table 1.** Serum TH Levels in NAFLD Patients

Thyroid Function Test	Mild NAFLD		Advanced NAFLD		Wilcoxon Rank Sum		Univariate Logistic Regression		Multivariate Logistic Regression	
	Discovery Cohort	Validation Cohort	Discovery Cohort	Validation Cohort						
TSH, μIU/mL, median	1.89 (Q1-Q3: 1.24–3.34)	2.37 (Q1-Q3: 1.55–3.29)	2.11 (Q1-Q3: 1.43–3.47)	2.66 (Q1-Q3: 1.43–3.47)	.590	.179	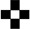 ^b^	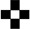	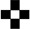	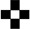
Free T4, ng/dL, median	1.15 (Q1-Q3: 1.01–1.35)	1.15 (Q1-Q3: 1.03–1.29)	1.25 (Q1-Q3: 1.06–1.33)	1.13 (Q1-Q3: 1.05–1.24)	.289	.734	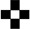	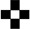	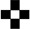	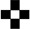
Free T3, pg/mL, median	3.70 (Q1-Q3: 3.20–4.00)	3.65 (Q1-Q3: 3.18–3.93)	3.40 (Q1-Q3: 3.10–3.70)	3.35 (Q1-Q3: 3.08–3.63)	.024^a^	.069	.025^a^	.074	.035^a^	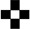
Reverse T3, ng/dL, median	27.45 (Q1-Q3: 22.48–36.60)	15.5 (Q1-Q3: 13.73–20.63)	39.6 (Q1-Q3: 28.00–47.33)	19.75 (Q1-Q3: 16.6–24.93)	.004^a^	.039^a^	.016^a^	.224	.032^a^	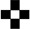
Free T_3_ to rT_3_ ratio, pg/mL per ng/dL, median	0.14 (Q1-Q3: 0.09–0.17)	0.23 (Q1-Q3: 0.16–0.28)	0.09 (Q1-Q3: 0.07–0.12)	0.18 (Q1-Q3: 0.13–0.21)	.002^a^	.013^a^	.005^a^	.016^a^	.005^a^	.019^a^
Free T_4_ to rT_3_ ratio, ng/dL per ng/dL, median	0.041 (Q1-Q3: 0.037–0.050)	0.072 (Q1-Q3: 0.05–0.08)	0.0327 (0.0278–0.0380)	0.057 (Q1-Q3: 0.04–0.07)	.002^a^	.025^a^	.014^a^	.033^a^	.027^a^	.057^a^

Abbreviation: Q, quartile. Two separate cohorts were evaluated independently: discovery cohort (n = 70 subjects total, 42 individuals with mild fibrosis and 28 individuals with advanced NAFLD); validation cohort (n = 60 subjects total; n = 30/group).

a Significant *P* values.

b Test not performed due to non significance on initial statistical analysis.

**Figure 6.**
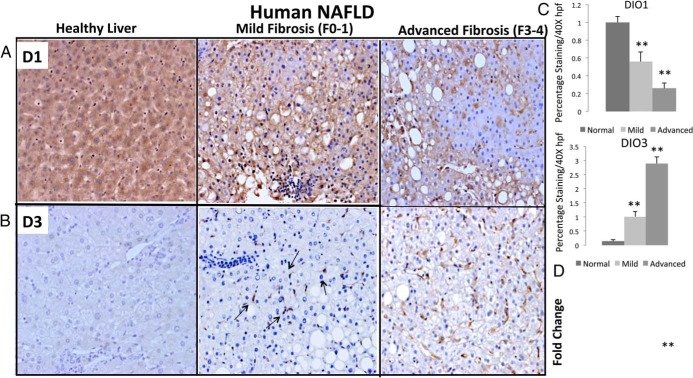
Changes in deiodinases and TH-responsive gene expression in humans with chronic liver injury. Immunohistochemistry for D1 (A) and D3 (B) in representative NAFLD patients with mild or advanced fibrosis (magnification, ×20). Morphometric analysis of staining in all subjects (controls, n = 4; mild fibrosis, n = 6; advanced fibrosis, n = 6). **, *P* < .01. C, Morphometric analysis of D1 and D3. **, *P* < .01. D, Microarray analysis of liver gene expression in the entire discovery cohort (n = 70 subjects) demonstrated the difference in *THRSP* expression between individuals with mild fibrosis (n = 42) and advanced fibrosis (n = 28). **, *P* < .01 after adjustment for multiple comparisons.

To assess the potential contribution of hepatic deiodinases to the differences in serum TH levels that we observed between mild and advanced NAFLD, we used IHC to compare expressions of D1 and D3 in the livers of a randomly selected subset of these NAFLD patients with either mild (F0–1; n = 6) or advanced (F3–4; n = 6) liver fibrosis and healthy controls without known liver disease (n = 4). Compared with healthy controls, NAFLD patients demonstrated reduced expression of D1 and increased expression of D3. In the mild NAFLD group, the intensity of D1 staining was generally less than observed in healthy controls, and rare patches of parenchyma that completely lacked hepatocyte D1 staining were noted in some patients. Moreover, in the group with advanced NAFLD, large areas of D1-negative hepatocytes were common (Figure 6, A and C). Conversely, both the intensity and extent of D3 staining increased in parallel with fibrosis stage in NAFLD patients, with consistently strong sinusoidal accumulation of D3-positive cells in all patients with advanced NAFLD fibrosis (Figure 6, B and C). Thus, like mice with BDL-induced liver fibrosis, humans with NAFLD-related liver fibrosis acquire a pattern of hepatic deiodinase expression that is reminiscent of developmental organogenesis. Because this D1/D3 switch is predicted to limit local exposure to T_3_, we evaluated hepatic expression of *THRSP* (a T_3_ responsive gene) in our entire NAFLD cohort. *THRSP* mRNA levels in patients with advanced NAFLD fibrosis were significantly lower than in patients with mild NAFLD fibrosis (Figure 6D), reflecting fibrosis-related changes in D1 and D3 and worsened intrahepatic hypothyroidism with advancing fibrosis (Table 1).

## Discussion

This study demonstrates, for the first time, that liver myofibroblasts and canonical Hedgehog signaling are key regulators of significantly contribute to intrahepatic changes in thyroid hormone homeostasis observed in a euthyroid sickness model after liver TH homeostasis during adult liver injury. These findings provide novel insight into both the mechanisms for, and the implications of, the euthyroid sick syndrome, showing that it is caused, in part, by stromal cell responses to tissue injury that limit exposure to T_3_, a factor that normally promotes cellular differentiation. Moreover, the results reveal that the liver fibrosis-associated euthyroid sick state is a mild form of peripheral tissue consumptive hypothyroidism mediated by the liver stromal cell up-regulation of D3 and reflected by detectable changes in serum levels of the D3 product rT_3_.

D1 and D3 are known to be differentially expressed during tissue injury (5–8). By studying mice and humans with fibrosing liver injury, we discovered that this reflects cell type-specific changes in deiodinase production. Specifically we showed that hepatocytes, the predominant D1-expressing cell type in healthy livers, down-regulate their expression of D1 during liver injury. In addition, we found that expression of this enzyme is also suppressed in various liver stromal cell types, whereas ductular cells accumulate D1. Moreover, we demonstrated that concomitant injury-related increases in D3 are caused by the up-regulation of *DIO3* expression in stromal cells and hepatic accumulation of such cells because hepatocytes remain predominantly D3 negative in chronically injured livers.

This switch from TH-activating to TH-deactivating enzyme predominance during liver fibrosis may have important implications for liver repair because D3 predominance has been noted in relatively undifferentiated tissues, including developing embryos and various cancers (3, 25, 26). The concept that increased D3 activity regulates tissue construction is further supported by evidence that knocking down D3 in adult zebrafish inhibited fin regeneration after amputation (27). Although the roles of TH during adult liver injury remain to be determined, it is noteworthy that significant up-regulation of D3 has been reported to occur acutely in rats after partial hepatectomy (5). In that study, however, concomitant suppression of *DIO1* was not observed. On the other hand, in rodents and humans with chronic liver injury and fibrosis, we demonstrated that D1 and D3 were reciprocally regulated and found that the net effect was increased accumulation of rT_3_ relative to active T_3_. Reduced hepatic exposure to T_3_ was accompanied by the suppression of TH-regulated gene expression, supporting the concept that local exposure to that important differentiating factor was limited. It is important to note that, although a majority of cells in advanced fibrosis remain hepatocytes, they represent a smaller proportion of liver cells per area secondary to an increase in nonparenchymal cell populations after injury. Therefore, it is possible that a small component of the 80% decrease in *THRSP* with advanced fibrosis in both rats and humans could be secondary to smaller proportions of hepatocytes per liver area.

Indeed, our work identified Hedgehog, an injury-induced signaling pathway, to be a key regulator of net hepatic deiodinase expression during liver injury. Treating cultured liver myofibroblasts with Hedgehog ligand increased their expression of *DIO3* mRNA. Conversely, targeted disruption of Hedgehog signaling in liver myofibroblasts of αsma-Cre/smo^flx/flx^ mice suppressed their myofibroblastic phenotype and not only prevented injury-related inhibition of D1 and induction of D3 in these cells but also abrogated loss of D1 expression in neighboring hepatocytes. The latter finding suggests that Hedgehog-responsive myofibroblasts ordinarily inhibit hepatocyte D1 expression during liver injury, possibly by limiting availability of T_3_. Further research is needed to clarify this. Nevertheless, the available data in experimental animals indicate that Hedgehog-responsive myofibroblasts are key determinants of hepatic deiodinase expression and thereby, local TH activity during liver injury.

Consistent with that concept, we found that intrahepatic concentrations of TH were higher after liver injury in mice that were depleted of Hedgehog-responsive myofibroblasts. Like mice, humans demonstrate Hedgehog pathway activation during cholestatic liver injury, NAFLD, and other forms of liver disease (28–30). Indeed, in both species, the level of Hedgehog pathway activity generally parallels the severity of myofibroblast accumulation and liver fibrosis (28). More significantly, in humans with an NAFLD-related liver injury but normal serum levels of T_4_, we demonstrated strong correlations between advanced liver fibrosis (a state of increased hepatic Hedgehog pathway activity and liver myofibroblast accumulation) (29), hepatic D3 induction and serum rT_3_ accumulation, suppression of intrahepatic T_3_ levels, and the inhibition of T_3_-responsive hepatic gene expression.

The aforementioned Hedgehog-directed changes in deiodinase activity might not be unique to injured liver. Dentice et al (24) demonstrated a *Gli2*-responsive promoter element in the *DIO3* gene and reported that *DIO3* expression is Hedgehog dependent in keratinocytes. This suggests that T_4_ might be preferentially converted to rT_3_, a relatively inert TH whenever Hedgehog pathway activity increases, thereby engendering localized pockets of peripheral tissue consumptive hypothyroidism in individuals with normal thyroid glands. Moreover, because the liver generates as much as 30%–50% of serum T_3_ (4), various types of liver disease that cause progressive liver fibrosis and sustained local hepatic activation of D3 might cause clinically significant systemic consumptive hypothyroidism. This possibility is supported by evidence that serum TSH elevations and frank hypothyroidism are common in many types of chronic liver disease (31, 32). An analogous situation has been reported to occur in young children with infantile hepatic hemangioendothelioma. In those individuals, D3-expressing tumor-associated stromal cells generate an enormous requirement for T_4_ that necessitates TH supplementation, and systemic hypothyroidism results when TH replacement is insufficient (33).

In summary, Hedgehog-regulated hepatic stromal cell responses that occur during adult liver repair shift the balance of local deiodinase expression to favor accumulation of biologically inert TH at the expense of biologically active TH. Although additional research is needed to clarify exactly how this process impacts liver repair, evidence suggests it might alter hepatic differentiation (3–5) as well as systemic TH homeostasis, thereby contributing to negative outcomes of fibrosing liver injury. As such, D3 may be a new therapeutic target in liver fibrosis, and its product, rT_3_, a novel biomarker of that process.
